# Model Stirrer Based on a Multi-Material Turntable for Microwave Processing Materials

**DOI:** 10.3390/ma10020095

**Published:** 2017-01-24

**Authors:** Jinghua Ye, Tao Hong, Yuanyuan Wu, Li Wu, Yinhong Liao, Huacheng Zhu, Yang Yang, Kama Huang

**Affiliations:** 1 College of Electronic and Information Engineering, Sichuan University, Chengdu 610065, China; 2015322050010@stu.scu.edu.cn (J.Y.); wuli1307@scu.edu.cn (L.W.); liaoyinhong2006@126.com (Y.L.); yyang@scu.edu.cn (Y.Y.); kmhuang@scu.edu.cn (K.H.); 2 College of Electronical Information Engineering, China West Normal University, Nanchong 637002, China; scu_mandela@163.com; 3 College of Information Science & Technology, Chengdu University of Technology, Chengdu 610000, China; Yuanyuanwu29@163.com

**Keywords:** materials processing, microwave heating, multi-material turntable, heating uniformity

## Abstract

Microwaves have been widely used in the treatment of materials, such as heating, drying, and sterilization. However, the heating in the commonly used microwave applicators is usually uneven. In this paper, a novel multi-material turntable structure is creatively proposed to improve the temperature uniformity in microwave ovens. Three customized turntables consisting of polyethylene (PE) and alumina, PE and aluminum, and alumina and aluminum are, respectively, utilized in a domestic microwave oven in simulation. During the heating process, the processed material is placed on a fixed Teflon bracket which covers the constantly rotating turntable. Experiments are conducted to measure the surface and point temperatures using an infrared thermal imaging camera and optical fibers. Simulated results are compared qualitatively with the measured ones, which verifies the simulated models. Compared with the turntables consisting of a single material, a 26%–47% increase in temperature uniformity from adapting the multi-material turntable can be observed for the microwave-processed materials.

## 1. Introduction

As a heating source, microwaves show many advantages over conventional heating methods, such as high heating efficiency, selective heating, easy controlling, and environmental friendliness. By virtue of these advantages, microwaves have been widely used to process materials in food engineering, chemical engineering, and many other fields [1,2,3,4,5]. For example, Liu et al. reported that good sterilization conditions can be observed under 2450 MHz microwave heating combined with 0.02 g·kg^−1^ ZnO nanoparticle addition [6]. Leadbeater et al. proposed that the preparation of biodiesel using microwave processing can result in a fast, easy route to this valuable biofuel with advantages of short reaction time, low oil/methanol ratio, and an ease of operation [7]. Lin et al. confirmed that the biodiesel yields from waste cooking oil can be improved by using microwave processing [8]. Roy et al. demonstrated that microwave processing is able to improve the modulus of Fe-Ni rupture by 60% than that of conventional samples [5].

However, there are still drawbacks limiting the large-scale industrial applications of microwave heating. Inhomogeneous heating is one of the major barriers [9]. When a high-power microwave is applied on the processed materials, the hot spots (huge temperature gradient at a given location) and thermal runaway (the uncontrollable temperature rise due to strong dielectric loss and temperature-positive feedback of the material) caused by microwave uneven heating may take place, which possibly leads to the damage of materials or even an explosion. To overcome this problem, several solutions have been proposed, like changing the position of processed sample, changing the composition of heated materials or adding a mode stirrer [10,11,12,13,14,15]. Among them, the last one is generally the most effective and employed widely.

Meanwhile, microwave ovens are commonly used applicators for processing materials. The turntable, as one of the most intuitive and common methods for increasing temperature uniformity, is used as a tray to carry and rotate the heated material in microwave ovens, so as to improve the temperature uniformity of microwave heating. Some studies related to the microwave oven turntable have been put forward. Geedipalli et al. proposed a discrete method to estimate the rotation of the turntable by rotating it 15° each time with the finite element method (FEM) [16]. The temperature uniformity contributed by such rotations was studied. Liu et al. proposed a FEM model to predict time-dependent temperature distributions of food samples with or without the rotation of turntable [17]. Pitchai et al. proposed a FEM model coupling electromagnetic and heat transfer equations to obtain the temperature distribution of a rotating multi-component meal. A microbial inactivation kinetics model for Salmonella Heidelberg was also studied to assess the food safety risk [18]. However, most of the studies mentioned above focused on the effect of position changes on heating uniformity. Actually, the turntable in microwave ovens can also play the role of mode stirrers when it is composed of different materials. It is supposed to be an economical and convenient way to access uniform temperature distribution in the process of material treatment.

**Figure 1:**
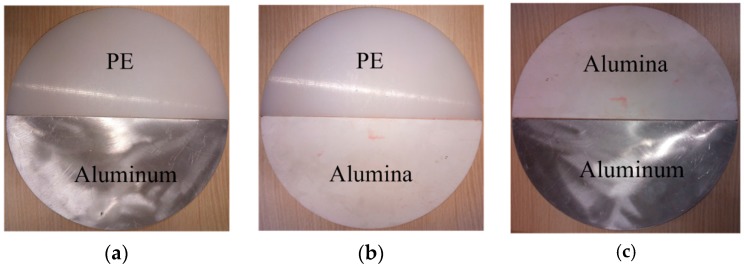
The proposed turntable structure and the corresponding experimental turntable. (**a**) PE-aluminum turntable; (**b**) PE-alumina turntable; and (**c**) alumina-aluminum turntable.

In this paper, a novel and simple multi-material turntable structure is first proposed to improve the heating uniformity of microwave processing materials effectively. Polyethylene (PE), alumina, and aluminum are selected as the material composition of turntable, as shown in Figure 1. The selection of these materials are random, because these materials are commonly used. Additionally, as long as the turntable is made of heterogeneous materials, the rotation can stir the electromagnetic field of microwave ovens. Multi-physics models are built and simulated to obtain the temperature distributions of processed samples with a constantly rotating multi-material turntable. The results are experimentally verified using an infrared thermal imaging camera and optical fibers. The heating uniformity of all the models are next analyzed by various criterions. The conclusion is drawn that multi-material turntables have great potential to improve microwave heating uniformity.

## 2. Methodology

### 2.1. Model Description

**Figure 2:**
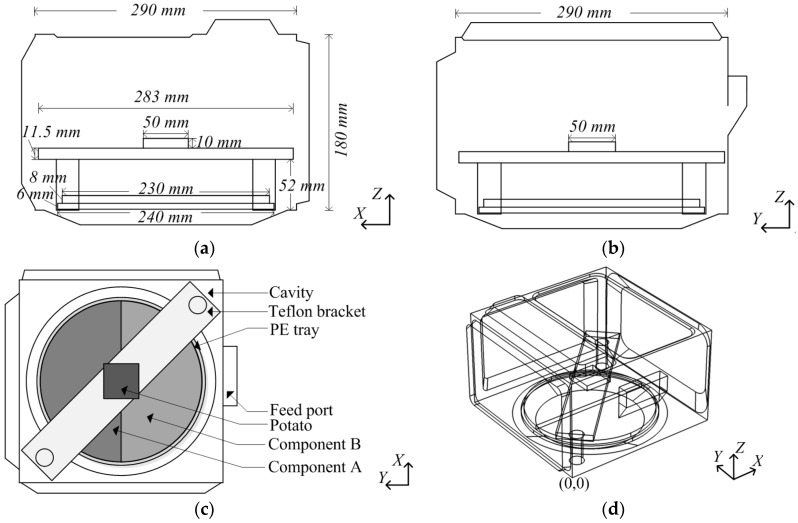
Geometry of the 3-D microwave oven: (**a**) *y*-*z* plane view; (**b**) *x*-*z* plane view; (**c**) *x*-*y* plane top view; and (**d**) 3D view.

The geometric model is developed based on a domestic microwave oven (P70D20TL-D4, Guangdong Galanz Enterprises Co., Ltd., Shunde, China) model with a rotating turntable composed of different materials. The geometric model consists of six components, including a cavity, a waveguide, a rotating turntable, a PE tray, a Teflon bracket and a heated sample, as shown in Figure 2. The waveguide and the cavity are filled with air. The turntable is formed by multiple materials, as shown in Figure 1, or a single material in different simulations, and it is placed on a PE tray. In order to observe the effect of the multi-material turntables on the heating uniformity, the heated potato slice is placed on the Teflon bracket, which is located above the center of the turntable. As shown in Figure 2d, the coordinate origin of the model is the lower right corner of the cavity. The rotation of the turntable in this applicator is continuous, covering the entire 360 degrees of rotation in 6 s. The waveguide is fed by a TE_10_ mode with an effective power of 300 W at a frequency of 2.45 GHz.

The initial temperature of the air, turntable and food inside the oven is 20 °C. The input parameters of the model are listed in Table 1. The thermal and dielectric properties of potatoes are obtained from the literature [4,19]. Considering the narrow temperature range, the thermal and electromagnetic parameters of the potatoes are treated as constant in the calculation.

### 2.2. Multi-Physics Calculation

Microwave-assisted processing of materials involves multiple physics, electromagnetic in the cavity and heated samples, as well as mass and heat transport.

#### 2.2.1. Governing Equation

The electromagnetic energy distribution inside an oven cavity is governed by the Maxwell’s waveform equation [20]:

(1) $$\nabla \times \mu_{r}^{-1}\times (\nabla \times E)-k_{0}^{2}(\varepsilon_{0}\varepsilon_{r}-j\sigma /\omega_{0})E=0$$

where *E* is the electric field, ε_0_ is the permittivity of vacuum, and ε_r_ is the relative permittivity of the medium. μ_r_ denotes the permeability, while ω_0_ represents the angular frequency. *k*_0_ is the wave number in a vacuum, and σ is the electrical conductivity.

Then, the power dissipated inside the potato can be obtained by the following equation [21,22]:

(2) $$P_{d}=\frac{1}{2}\omega_{0}\varepsilon_{0}\varepsilon ʺ{\left|E\right|}^{2}$$

where ε″ is the imaginary part of relative permittivity of potato. The dissipated power term is the heat source in transient heat transfer of potato domain [23,24].

(3) $$\rho C_{p}\frac{\partial T}{\partial t}=k\nabla^{2}T+P_{d}$$

where ρ, *C*_p_ and *k* are the density, heat capacity and thermal conductivity of potatoes, respectively.

#### 2.2.2. Boundary Condition

##### ● *Electromagnetic boundary condition*

The metallic waveguide and cavity walls are considered as perfect electric conductors, where the following boundary condition applies [25]:

(4) $$E_{\mathrm{tangential}}=0$$

##### ● *Heat transfer boundary condition*

In this model, the upper surface of the potato slice exchanges heat with air by convection, expressed as [26]:

(5) $$-k\cdot \partial T/\partial n=h\cdot (T-T_{\mathrm{air}})$$

where ∂*T*/∂*n* is the gradient of the temperature perpendicular to the temperature field interface, *T*_air_ is the temperature of the air inside the oven, and *h* is the heat transfer coefficient with the value of 10 W/(m^2^∙K), which is a typical value used for natural convective heat transfer in air [18]. The thermal boundaries between potato slices and the Teflon bracket are set as insulators.

### 2.3. Continuous Algorithm for Turntable Rotation

**Figure 3:**
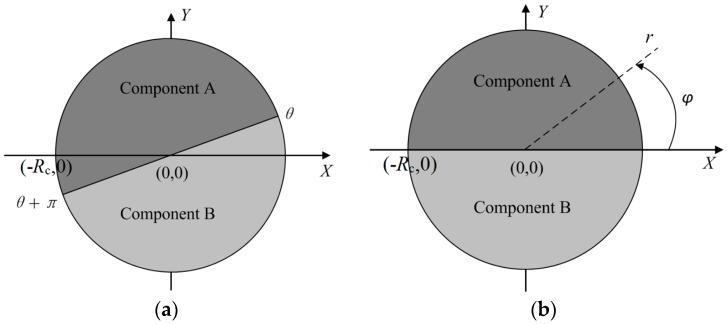
The turntable in a two dimensional cylindrical coordinate system (dark gray represents Component A, light gray represents Component B). (**a**) The initial position of the turntable; (**b**) The position of the turntable during rotation.

To simulate the heating process, we adopt a continuous algorithm, which can avoid re-meshing during the calculation process, so as to obtain accurate results. In this algorithm, the permittivity of the rotating region is set as a function of space and time to represent the rotation of the multi-material turntable. As for this model, the rotating region is turntable region. Since the turntable is rotating on the *x*-*y* plane, it can be treated as a two-dimensional cylindrical coordinate system. The components of the multi-material turntable are expressed as Component A and Component B for short, as shown in Figure 3a. It can be noted that all the mesh dots inside the turntable region satisfy the following constraint condition:

(6) $$r<R_{c}$$

where *R*_c_ is the radius of the turntable, and *r* is the distance from the mesh dots to the origin (0,0). At the initial moment, mesh dots inside Component A also satisfy the constraint condition as:

(7) 0<φ<π

To represent constraint conditions change with the actual motion of the turntable, the constraint condition in Equation (7) is rewritten as:

(8) θ<φ<θ+π

where θ is the starting central angle of Component A, as shown in Figure 3b. θ is a function of time, which can be expressed as:

(9) θ=t⋅dθ

where *d*θ is the rotating angle of the turntable in one second. By using the step function with Equations (6)–(9), the mesh dots inside Component A can be expressed as:

(10) $$\Phi_{A}=step(R_{c}-r)\cdot step(\varphi -td\theta )\cdot (td\theta +\pi -\varphi )$$

where *step*() represents the step function. Then the permittivity of the turntable during the rotating process can be expressed as:

(11) $$\varepsilon_{t}=\Phi_{A}\cdot \varepsilon_{A}+(1-\Phi_{A})\cdot \varepsilon_{B}$$

where ε_A_ and ε_B_ is the permittivity of Component A and Component B, respectively. If the electrical conductivity of the Component A differs from that of Component B, it can also be expressed in a similar form. By solving Equation (1) with a time-varying permittivity ε_t_, the electric field distribution in each time step can be obtained without re-meshing during the calculation. Then, the electromagnetic power dissipation is input as a heat source term into the heat conduction equation to obtain the temperature distribution.

The time step is set as 0.2 s in this work, and the corresponding rotating angle *d*θ in each time step is 12°. The heating process is computed by the multi-physics software COMSOL Multiphysics (V5.1, COMSOL Inc., Stockholm, Sweden).

### 2.4. Experiment Setup

**Figure 4:**
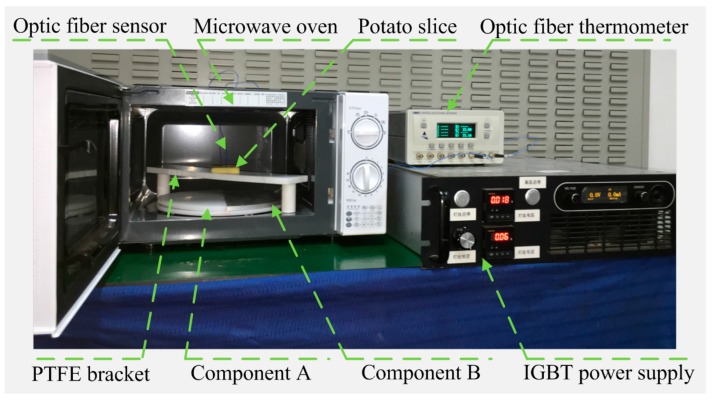
The photo of experimental microwave heating system.

**Figure 5:**
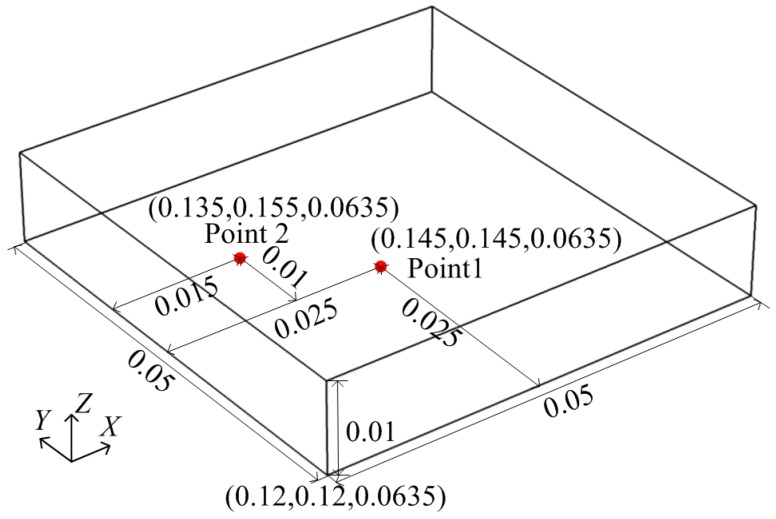
Position of the fiber-optic sensors (unit: m).

An experimental system is set up to validate the proposed model, as shown in Figure 4. A potato slice is subjected to microwave heating for 12 s. Two fiber-optic sensors are used to record the temperature changes of two points. The temperature sensor, with accuracy of 0.1 °C (FISO FOT-NS-967A, FISO Technologies, Quebec, QC, Canada), is connected to a FISO OSR-4 workstation. One sensor is inserted into the center of the bottom, inside the potato, and the other into an upper left point of the potato bottom (Point 2 in Figure 5) to record temperature profiles at every second during heating. Measured data is analyzed using digital software, FISO Command WorkStation (v1.10.9; FISO Technologies, Quebec, QC, Canada). Once completing microwave heating, the potato slice is taken out of the microwave oven. Its temperature distribution on the top surface is captured by an infrared imaging camera with the accuracy of 0.03 K (VarioCAM hr inspect 500, InfraTec., Dresden, Germany). The insulated gate bipolar transistor (IGBT) power supply (DDY10-5K/0V8-S220/F02, Sichuan Injet Electric Co., Ltd., Deyang, China) is employed as a high voltage DC power supply for a magnetron to stabilize the microwave frequency, due to its small high voltage output ripple.

## 3. Results and Discussion

### 3.1. Experimental Validation

**Figure 6:**
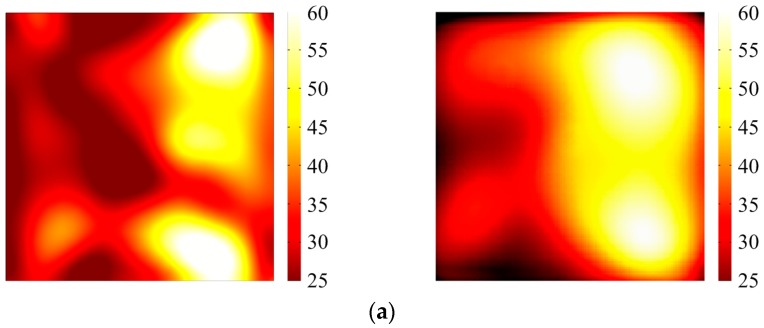

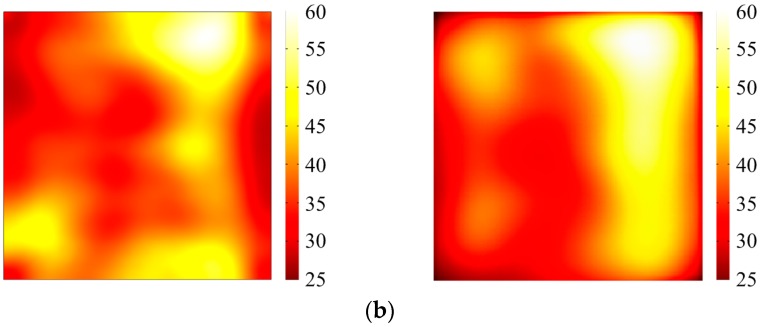
Comparisons of experimental and simulated spatial temperature profiles of potatoes subjected to 12 s heating in a 300 W microwave oven (the simulation results are on the left, and the experimental results are on the right (unit: °C): (**a**) the turntable is composed of PE; and (**b**) turntable is composed of PE and alumina.

Since the simulation cases in this paper are similar, two typical cases with single-material (PE) and multi-material (PE-alumina) turntables are picked to verify the theoretical model in experiments. Simulated and measured temperature distributions of the potato surface are presented and compared in Figure 6. It is obvious that the locations of cold and hot spots in simulations and experiments are roughly the same. Given the fact that water evaporation is not considered, a good match is obtained between the experimental and simulated results.

**Figure 7:**
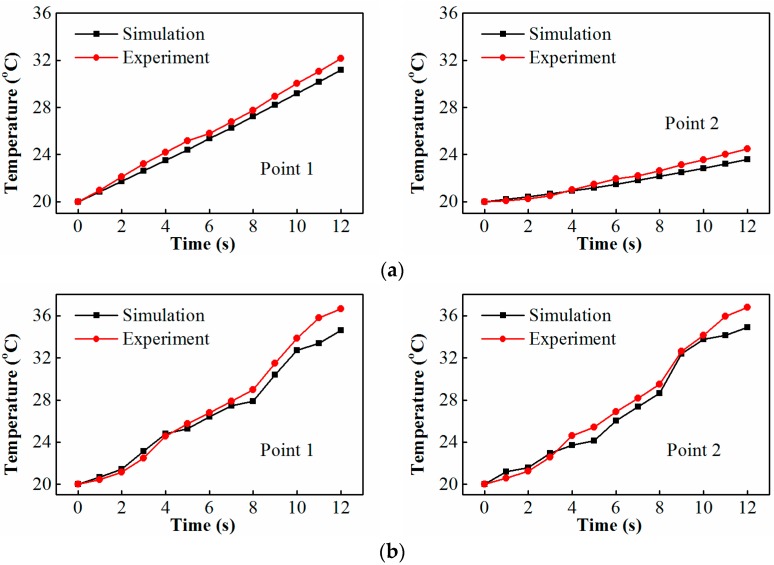
Simulated and experimental temperature profiles at two points of the potato subjected to 12 s heating in a 300 W microwave oven (refer to locations in Figure 5): (**a**) the turntable is composed of PE; and (**b**) turntable is composed of PE and alumina.

Measured transient temperature profiles at two points are compared with the simulated ones, as shown in Figure 7. One can observe from Figure 7 that the simulation data agree well with the experimental results. The small deviation existed in the comparisons may be due to the use of assumed constant electrical and thermal parameters.

### 3.2. Uniformity Analysis

#### 3.2.1. Variations of Electric Field

**Figure 8:**
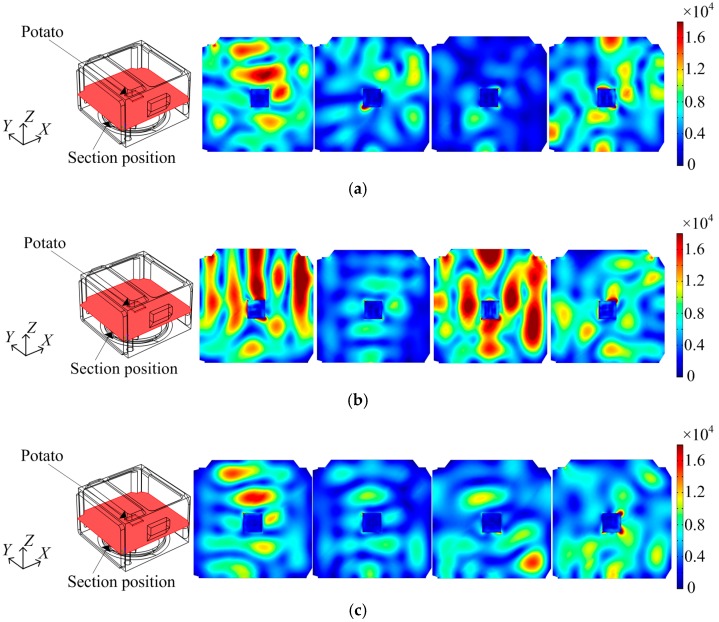
Electric field distributions of horizontal sections (*Z* height: 0.0685 m) of the microwave oven with the turntable rotation angles of 0°, 90°, 180°, and 270° (unit: V/m): (**a**) PE-aluminum turntable; (**b**) PE-alumina turntable; and (**c**) alumina-aluminum turntable.

The rotation of the multi-material turntable provides mode stirring, which makes the electric field in the microwave oven change with time, even though all of the other parameters are fixed. In order to observe the effect of the rotation of a multi-material turntable on the electric field distribution, the electric field distributions of the center horizontal section of the microwave oven with the turntable rotation angles of 0°, 90°, 180°, and 270° of the turntable are presented in Figure 8. The position of the section is also shown in Figure 8. We can clearly see that the electric field distribution in the microwave oven varies significantly with time. The position of a strong electric field at a certain moment is likely to shift largely at the very next moment. The variation of the electric field will be helpful to the improvement of heating uniformity.

#### 3.2.2. Hot Spot Analysis

**Figure 9:**
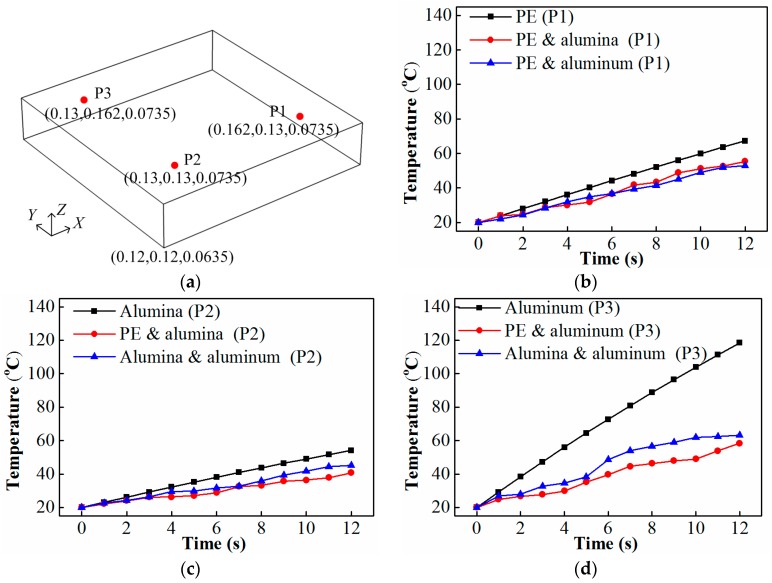
Hot spot analysis: (**a**) location of P1, P2, and P3 (unit: m); (**b**) temperature rise curve of P1; (**c**) temperature rise curve of P2; (**d**) temperature rise curve of P3.

Here, the temperature rise curves at hot spots of multi-material turntables are compared with those of single material turntables. In this case, when the turntable is composed of only PE, P1 (Figure 9a) is one of the hot spots in the potato. Its temperature change during the heating process is plotted in Figure 9. To check whether it is still a hot spot under the other cases, the temperature rise curves of P1 with multi-material turntables are also presented in Figure 9. Following the same principle, the temperature rise curve of P2 (one of the hot spot when the turntable is composed of only alumina) and P3 (one of the hot spot when the turntable is composed of only aluminum) in the heating process of turntables composed of single and multiple materials are also provided in Figure 9.

From Figure 9, one can conclude that, for the case where the turntable is composed of multiple materials, the temperature rise in the hot spot becomes much smaller, in comparison with the case with the single material turntable. This means that turntables composed of multiple materials can eliminate the hot spots that exist in the heating process of a single-material turntable. Although it may generate new hot spots, the non-uniform heating will be greatly reduced due to the variation of the electric field in the heating process.

#### 3.2.3. Quantification of Heating Non-Uniformity

In order to quantify the non-uniformity of temperature, the coefficient of variation (COV), namely the ratio of the standard deviation to the mean, is adopted because it can remove the effect of the mean on the standard deviation and it is widely used to quantify the heating non-uniformity [16,18]. The related statistics of the turntable composed of multiple materials or a single material are listed in Table 2. It shows that when the turntable is composed of only one material, the mean temperature rise of the potato may be very small (e.g., with the alumina turntable, the mean temperature rises by 9.20 °C). However, when the turntable consists of multiple materials, the mean temperature rise of the potato can approach approximately 20 °C, which indicates that the microwave energy is effectively absorbed. Additionally, the COV shows that temperature uniformity of the potato, when the turntable is composed of PE and alumina, increases at least (0.625–0.332)/0.625 or 47% than that of alumina. Similarly, when the turntable is made of PE and aluminum, its improvement on temperature uniformity achieves 46% ((0.585–0.317)/0.585) compared to that of aluminum. However, the optimization in the temperature distribution with the aluminum-alumina turntable only attains 29% ((0.585–0.413)/0.585). The comparisons indicate that the multi-material turntable can improve the heating uniformity effectively and the amplitude of the improvement is related to the turntable components.

In many cases, one may be more interested in the temperature extremes than an equally-weighted standard deviation. For example, with consideration of microbial destruction, the processed food materials need to reach a minimum temperature for safety [16]. Thus, the range of temperatures is an important parameter to characterize temperature non-uniformity in the processed sample. Table 3 gives the quantified non-uniformity data of the potatoes in terms of the range at the end time (12 s). In Table 3, the 10th percentile refers to the temperature in the top 10% highest positions when the sample temperatures of all the mesh dots are arranged from low to high. Similarly, the 90th percentile refers to the temperature in the top 90% highest positions. For example, when the sample has ten mesh dots, the temperatures of these ten mesh dots are arranged from low to high as 1 °C, 2 °C, 10 °C, the 10th and 90th percentile in this case are 1 °C and 9 °C, respectively. The difference in the 10th and 90th percentile values is considered to give a true representation of the sample temperature range [16]. The difference over the rise (similar to COV) is an alternative definition of quantifying non-uniformity of temperature. The smaller it is, the more concentrated the temperature range of the material, namely the better the temperature uniformity. Improvements in the three corresponding comparisons presented in the COV section above are, respectively, 43%, 43%, and 26%, calculated by the difference over the rise.

**Figure 10:**
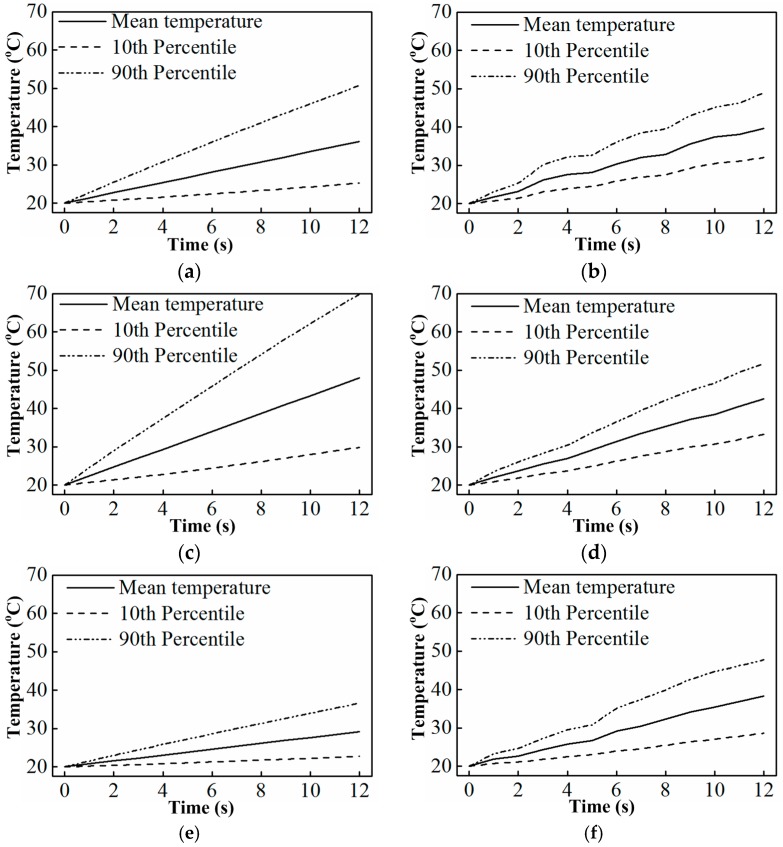
Potato temperature range as a function of heating time with a multi-material turntable or a single material turntable: (**a**) PE turntable; (**b**) PE-alumina turntable; (**c**) aluminum turntable; (**d**) PE-aluminum turntable; (**e**) alumina turntable; and (**f**) alumina-aluminum turntable.

Figure 10 illustrates the middle 80 percentile potato temperature range as a function of heating time with a turntable composed of multiple materials or a single material. From these figures, we can clearly see that the potato temperature range increases with the mean temperature rise. Additionally, the potato temperature range increases more rapidly in the case of a single material turntable as compared to that of the multi-material turntable (except Figure 10e, whose temperature rise is too small when compared with the others), which means the temperature distribution in the case of a multi-material turntable is more concentrated than that of a single material turntable. In other words, it means that the sample temperature distribution in the case of a multi-material turntable is more uniform than that of a single material turntable. This further demonstrates the aid of the multi-material turntable in improving the heating uniformity of the processed sample.

## 4. Conclusions

In this paper, a coupled model to simulate the heating process with rotating turntables in a microwave oven is provided and the effect of turntable components on the heating uniformity is studied. Experiments are also conducted to verify the accuracy of the simulated model. Several conclusions can be drawn from our research:

(1) The computational results from the proposed model are in good agreement with the experiment. It shows that this model can serve as an effective method to deal with the thermal analysis with rotating turntables during microwave heating.
(2) By using various quantitative analysis methods, it can be concluded that multi-material turntables can improve the temperature uniformity of the processed material (increase range: 29%–47%). The amplitude of the improvement, however, is related to the composition of the turntable.
(3) The work will be helpful to optimize the heating status of the existing microwave oven for processing materials.

## Figures and Tables

**Table 1:** Summary of material properties applied in the model.

Parameter	Domains	Value
Dielectric constant (ε′)	Air	1
	Alumina	9
	PE	2.3
	Aluminum	1
	Teflon	2.1
	Potato	57
Dielectric loss factor (ε′′)	Potato	17
	Others	0
Specific heat capacity (*C*_p_, J/(kg·K))	Potato	3640
Density (ρ, kg/m^3^)	Potato	1050
Thermal conductivity (*k*, W/(m^3^·K))	Potato	0.648
Heat transfer coefficient (*h*, W/(m^2^·K))	Potato–Air	10
Electrical conductivity (S/m)	Aluminum	3.774 × 10^7^
	Others	0

**Table 2:** Non-uniformity, as calculated by COV with 2919 mesh dots, in potato temperature distribution after 12 s heating with a turntable composed of multiple materials or a single material.

Parameter	PE	Alumina	Aluminum	PE and Alumina	PE and Aluminum	Alumina and Aluminum
Mean Temperature Rise (°C)	16.13	9.20	27.99	19.64	22.46	16.86
Standard Deviation (°C)	10.80	5.75	16.37	6.52	7.11	6.96
COV	0.670	0.625	0.585	0.332	0.317	0.413

**Table 3:** Non-uniformity, as calculated using 10th and 90th percentiles (see text for definition) with 2919 mesh dots, in potato temperature distribution after 12 s heating with a turntable composed of multiple materials or a single material.

Parameter	PE	Alumina	Aluminum	PE and Alumina	PE and Aluminum	Alumina and Aluminum
10th Percentile (°C)	25.20	22.74	29.86	32.02	33.24	27.93
90th Percentile (°C)	50.88	36.59	69.91	48.97	51.66	45.9
Difference (°C)	25.68	13.85	40.05	16.95	18.43	17.97
Mean Temperature Rise (°C)	16.13	9.20	27.99	19.64	22.46	16.86
Difference/Rise	1.589	1.505	1.432	0.863	0.820	1.066
